# Step-by-step ligation of the internal iliac artery

**DOI:** 10.4274/jtgga.galenos.2018.2018.0124

**Published:** 2019-05-28

**Authors:** İlker Selçuk, Bora Uzuner, Erengül Boduç, Yakup Baykuş, Bertan Akar, Tayfun Güngör

**Affiliations:** 1 Clinic of Gynecologic Oncology, Health Sciences University, Zekai Tahir Burak Woman’s Health Training and Research Hospital, Ankara, Turkey; 2 Department of Anatomy, Kafkas University Faculty of Medicine, Kars, Turkey; 3 Department of Obstetrics and Gynecology, Kafkas University Faculty of Medicine, Kars, Turkey; 4 Clinic of Obstetrics and Gynecology, VM Medical Park Kocaeli Hospital, Kocaeli, Turkey

**Keywords:** Gynecologic, hypogastric, bleeding, postpartum, pelvic

## Abstract

The internal iliac artery is the main vascular supply of pelvic visceral structures. All pelvic surgeons must know the anatomic landmarks and basic steps of internal iliac artery ligation in order to stop massive pelvic hemorrhage. This cadaveric demonstration and clinical review of the internal iliac artery shows the anatomic landmarks and basic steps of internal iliac artery ligation.

## Introduction

The anatomy of the internal iliac artery (IIA) has been well documented previously and it is the major blood supply of pelvic structures. It arises from the common iliac artery and runs infero-medially in the pelvis. An enormous number of small vessels, collateral circulation, and variations exist in pelvic vasculature (1,2). 

The role of IIA ligation to control intractable pelvic hemorrhage  has been described by Kelly (3) for the first time in 1893 for a cervical carcinoma case. Ligation of the IIA could also be a life-saving procedure during peripartum bleeding (4,5). In selected cases, ligation of IIA is also an option during intraperitoneal bleeding where the exact location could not be identified because IIA is the main blood supply of the pelvic viscera (6). During a massive pelvic hemorrhage or peripartum bleeding, bilateral ligation of the IIA reduces the pelvic arterial blood flow by 49% and pulse pressure by 85% (7). After bilateral ligation of IIA in the long term period, the collateral circulation will maintain the re-functioning of the IIA. The deep femoral artery is the principal vascular supply to provide re-vasculature to the IIA. Anastomosis between the medial femoral circumflex and obturator artery, and the lateral femoral circumflex and superior gluteal artery are the main connection areas (8). Additionally, the ovarian artery also provides blood flow to the uterus. Despite bilateral ligation of the IIA, future reproductive potential is not affected totally and term pregnancies have also been reported in the literature (9,10).

Despite some technical difficulties with regard to anatomic relationships and potential complications,  ligation of IIA provides a rapid way to decrease the pelvic arterial blood flow. This clinical and photographic review shows the step-by-step surgical technique used in ligation of the IIA because all pelvic surgeons need to know how to ligate the IIA.

## Material and Methods

The figures of this study were obtained during cadaveric dissections at the Consultants in Obstetrics and Gynecology-Management of Peripartum Bleeding and Morbidity Cadaveric Course, Bahçeşehir University Faculty of Medicine, İstanbul, 2017, and the Management of Peripartum Hemorrhage Cadaveric Course, Kafkas University Faculty of Medicine, Kars, 2018.

### Probable indications of internal iliac artery ligation

Ligation of the IIA has a proven success rate in controlling massive pelvic hemorrhage, varying between 40% and 100%, and obstetric pathologies occupy the first place as the leading factor (11,12). Table 1 shows the obstetric and gynecologic indications of IIA ligation.

### Probable complications during ligation of the internal iliac artery

The risk of operative injury beyond success is the major gap beneath the feasibility of the procedure (Table 2). A detailed knowledge of the anatomy is required along with good exposure to achieve the procedure with the maneuver of traction and counter-traction. Bilateral ligation of the IIA is better to control the total blood flow in the pelvis and the surgeon would prefer to change the operative side for a comfortable surgical practice.

### Basic anatomy of the internal iliac artery

**Figure 1:**
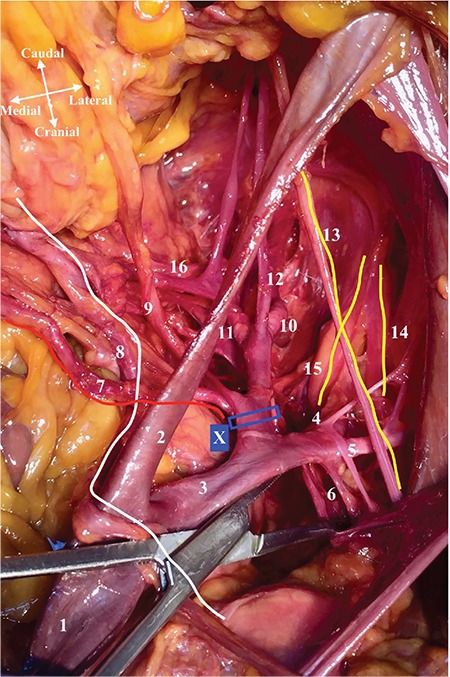
Anatomy of the internal iliac artery. Basic anatomic structures and branches of the internal iliac artery; Right pelvic side wall, superior view (1. Common iliac artery, 2. External iliac artery, 3. Internal iliac artery (IIA), 4. Superior gluteal artery, 5. Iliolumbar artery, 6. Lateral sacral artery, 7. Uterine artery (red line), 8. Ureter (white line), 9. Umbilical artery (obliterated), 10. Inferior gluteal artery, 11. Internal pudendal artery, 12. Obturator artery, 13. Obturator nerve (yellow line), 14. Lumbosacral trunk (yellow line), 15. S1 Nerve (yellow line), 16. Middle rectal artery, X. Ligation point of IIA)

The aorta is divided into left and right common iliac arteries at the level of the fourth and fifth lumbar vertebra (L4-5) and after a pathway of 4.0-5.0 cm the common iliac artery gives the branches of external iliac artery and IIA. The IIA runs infero-medially after the pelvic brim (Figure 1) and has two divisions, posterior and anterior (Table 3). The anterior division starts after 3.5-5.0 cm from the origin of the IIA and branches of the posterior division diverge before that part. The anterior division is the main blood supply of the pelvic viscera.

Accurate identification of adjacent anatomic structures (Table 4) will make the procedure easier and decrease the risk of complications during the surgical approach (13).

### Clinical tips

Monopolar or bipolar electrocoagulation could also be used during dissection of the surgical field, bleeding from small veins will stop spontaneously; nevertheless, care must be taken. Before ligation of the IIA, dissection of the ureter is extremely important and inspection is better than just palpation of the ureter in the context of preventing any probable injury. Although ligation of IIA could be performed at any side of the patient either right or left, the surgeon must be careful during dissection and traction of the IIA because movement of the right-angle clamp from medial to lateral will cause a laceration on the external iliac vein.

## Figures and Tables

**Table 1:**
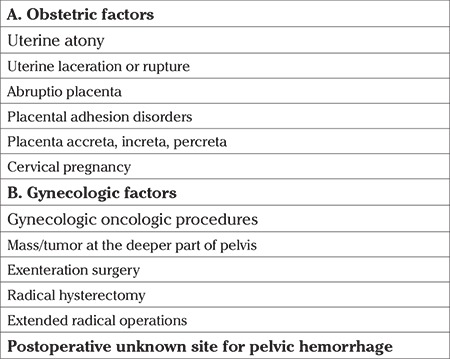
Indications of internal iliac artery ligation

**Table 2:**
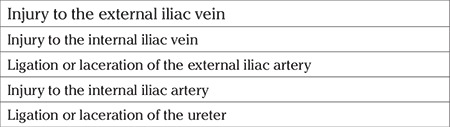
Operative complications during ligation of internal iliac artery

**Table 3:**
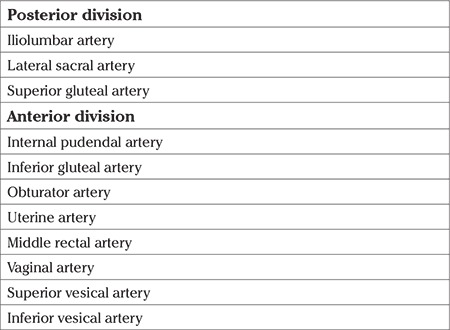
Branches of internal iliac artery with regard to divisions

**Table 4:**
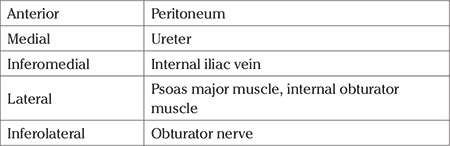
Anatomic relations of internal iliac artery

**Figure 2:**
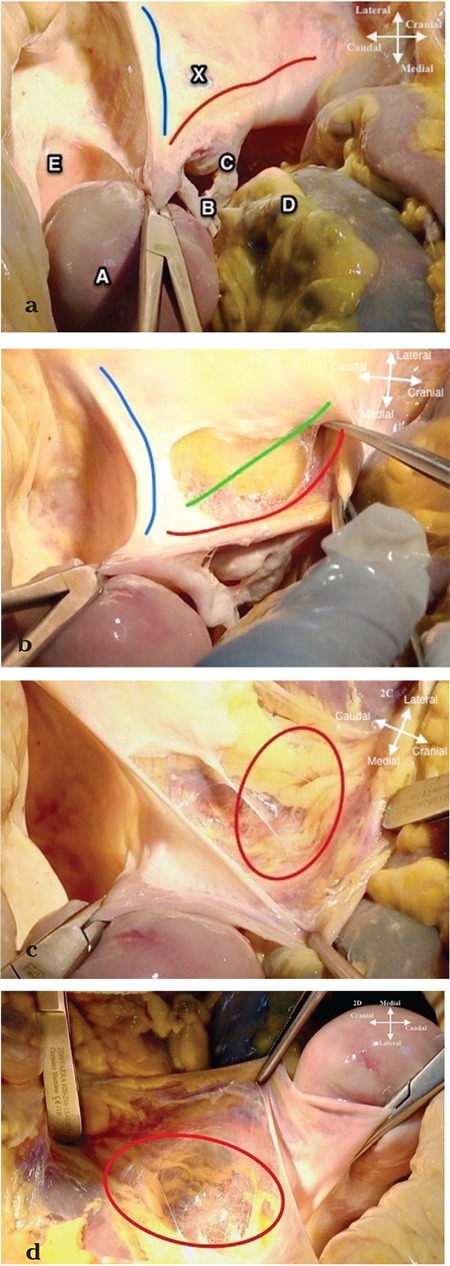
Entering the retroperitoneum (2a-d). a. The lateral parietal peritoneum over the pelvic side wall (over the psoas major muscle and external iliac artery) between the round ligament (ligamentum teres uteri) (blue line) and infundibulopelvic ligament (ligamentum suspensorium ovarii) (red line) is cut (X). During this step the uterus is pulled towards the counter side (caudally) of the pelvic wall where we plan to enter the retroperitoneum. (A: Uterus, B: Right fallopian tube, C: Right ovary, D: Rectum, E: Bladder), b. The incision is extended cranially to the level of pelvic brim (green line) parallel to the infundibulopelvic ligament, c. superior view, d. lateral view: Posterior leaf of the broad ligament (ligamentum latum uteri), (the peritoneum with the ovarian vessels), is retracted medially so the retroperitoneal area (red circle) is visualized

**Figure 3:**
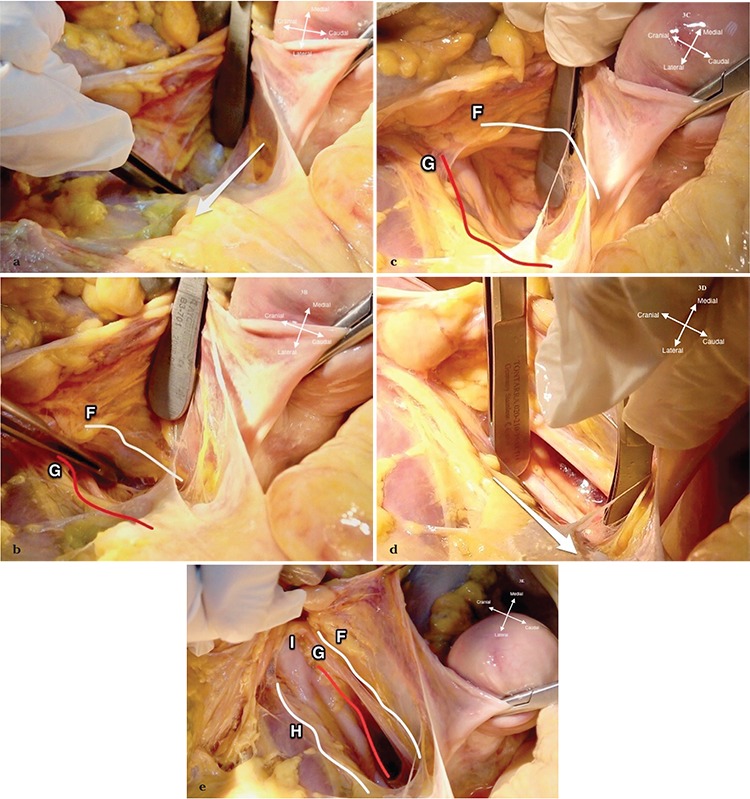
Identification of the ureter and internal iliac artery (3a-e). a. The ureter runs on the posterior leaf of the broad ligament under the ovarian vessels, medial to the anterior branch of internal iliac artery; therefore, holding the posterior leaf and making a blunt dissection towards sacrum (white arrow) targeting the deeper part of posterior leaf will guide to identify the ureter; b, c. The ureter (F, white line) is identified on the base of the broad ligament, medial to the internal iliac artery (G, red line); d, e. The adipose and lymphatic tissue over the internal iliac artery is dissected with a caudal movement (white arrow) (3d). The ureter (F, white line), internal iliac artery (G, red line), external iliac artery (H), and the common iliac artery (I) will be noticed just over the pelvic brim at the upper part of pararectal space (3e) [borders of pararectal space: posteriorly sacrum, medially ureter and rectum, laterally internal iliac artery and anteriorly uterine artery and cardinal ligament (ligamentum transversum cervicis)]

**Figure 4:**
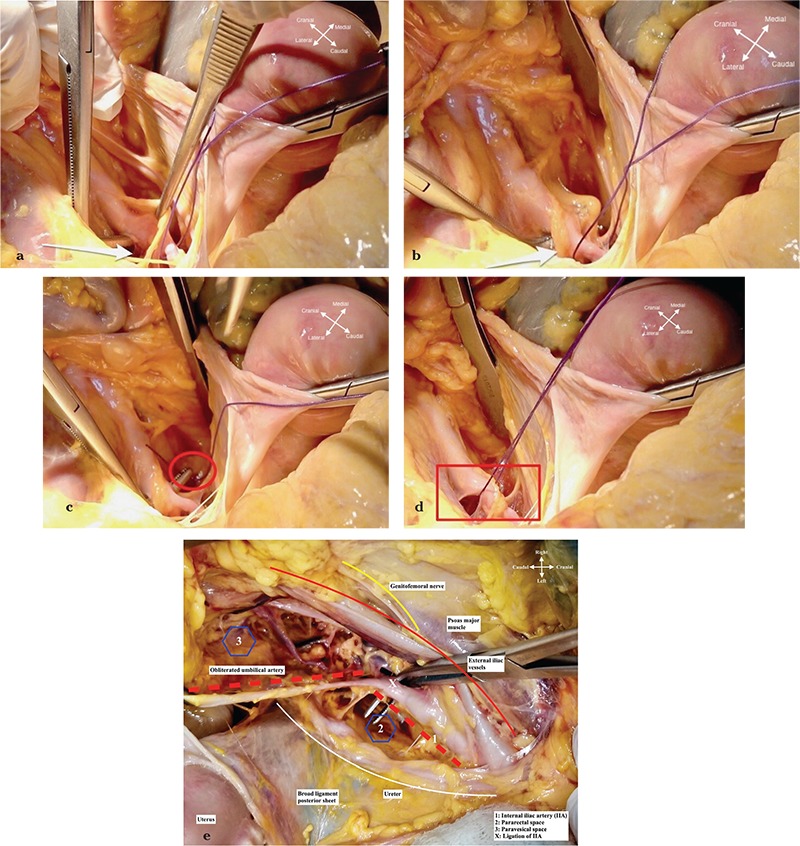
Ligation of internal iliac artery (4a-e). a. A right-angle clamp is placed under the anterior division of internal iliac artery (white arrow), after the main trunk gives the branches of the posterior division (3.5 cm after the origin of internal iliac artery); b. Care should be taken not to harm the underlying external iliac vein, located on the infero-lateral part of the internal iliac artery. Accordingly, the right-angle clamp should be moved from lateral to medial under the internal iliac artery (white arrow) while holding the end point of clamp upperly; c. After getting on the other side beneath the internal iliac artery, the suture material is grasped (red circle) and pulled backwards in the same direction; d. The ureter, external iliac artery, and other important anatomic landmarks are re-checked and finally the suture is tied carefully (red rectangle); e. Superior view of right pelvic side wall, how to ligate the internal iliac artery, with close anatomic structures
